# Clinical and radiological comparison of the zero-profile anchored cage and traditional cage-plate fixation in single-level anterior cervical discectomy and fusion

**DOI:** 10.1186/s40001-022-00813-w

**Published:** 2022-09-30

**Authors:** Jun Zhang, Shanxi Wang, Xiangyu Tang, Wei Xiong, Hua Wu, Chaoxu Liu, Feng Li

**Affiliations:** 1 Department of Orthopedics, Tongji Hospital, Tongji Medical College, Huazhong University of Science and Technology, Jiefang Avenue 1095, Wuhan, 430030 People’s Republic of China; 2 Department of Orthopedics, The First Affiliated Hospital of Kunming Medical University, Kunming Medical University, Xichang Road 295, Kunming, 650000 People’s Republic of China; 3 Department of Radiology, Tongji Hospital, Tongji Medical College, Huazhong University of Science and Technology, Jiefang Avenue 1095, Wuhan, 430030 People’s Republic of China

**Keywords:** Zero-profile, Cage, Anterior cervical discectomy and fusion, Dysphagia

## Abstract

**Background:**

The aim of this study was to compare the clinical outcomes and radiographic parameters of the zero-profile anchored cage and traditional cage-plate fixation in single-level anterior cervical discectomy and fusion (ACDF).

**Methods:**

Between January 2016 and November 2018, a total of 68 patients with degenerative cervical spondylosis who underwent single-level ACDF were evaluated in this retrospective study. Thirty-five patients were treated with the zero-profile anchored cage (Zero-P group), and 33 patients were treated with the traditional cage-plate fixation (Cage group). The two groups were compared in reference to clinical outcomes and radiographic parameters.

**Results:**

The mean operation time in the Zero-P group was significantly shorter than that in the Cage group. The incidence of postoperative dysphagia in the Cage group was higher than that in the Zero-P group at 3 months and 12 months postoperatively. No bony spurs were found in the Zero-P group, whereas 5 patients in the Cage group developed bony spurs. There were no statistically significant differences between the two groups in the JOA scores, VAS scores, NDI scores, C2-7 Cobb angles, segmental Cobb angles, total interbody height or fusion rates at 3 months or 12 months postoperatively.

**Conclusion:**

In this study, both the zero-profile anchored cage and traditional cage-plate fixation were demonstrated to be effective and safe strategies. Given the lower incidence of dysphagia and degenerative changes, zero-profile anchored cage is a good option.

## Background

Cervical spondylosis is a type of neurological disease caused by the stimulation or compression of the adjacent spinal cord due to degeneration of the cervical spine, and common symptoms include neck and arm pain associated with radiculopathy or myelopathy [1, 2]. Based on relevant clinical symptoms and evidence of root or spinal cord compression on recent radiographic examinations, surgical intervention is indicated as follows: (1) the clinical symptoms are severe and progress rapidly, seriously affecting the normal life of the patient; and (2) conservative treatment is ineffective [3]. Since the 1950s, anterior cervical discectomy and fusion (ACDF), as a standard technique, has been widely used in patients with cervical spondylosis. Some studies have indicated that ACDF with a cervical plate and cage results in a more satisfactory fusion rate and lower failure rate [4] but a higher incidence of plate-associated complications, such as postoperative dysphagia [5–7].

To prevent these problems, zero-profile anchored cage (Zero-P) has been recommended by some researchers. Compared with other technologies, Zero-P has the advantage of providing immediate stability. Furthermore, many reports have shown that it is a promising strategy to reduce the occurrence of postoperative dysphagia [8–10]. The purpose of this study was to compare the clinical and radiological outcomes of patients receiving a zero-profile anchored cage with those using a traditional cage-plate fixation.

## Materials and methods

### Patients

After the approval of our institutional review board, a total of 68 patients were enrolled in this study from January 2016 to November 2018. All patients were treated with ACDF. The indications for surgical treatment in each patient included failure of nonsurgical treatment or progressive neurologic dysfunction combined with instability or deformity of the cervical spine. Thirty-three patients received the conventional cage-plate fixation (Cage group), and 35 patients received the zero-profile anchored cage (Zero-P group). There were no statistically significant differences between the two groups in terms of age, gender, surgical site, preoperative functional scores or preoperative radiographic outcomes (Table 1).

**Table 1:** Baseline characteristics of the study population

	Cage group (*n* = 33)	Zero-P group (*n* = 35)	*P* value
Age (years)	47.8 ± 11.0	49.6 ± 9.1	0.583
Gender (male/female)	17/16	20/15	0.808
Surgical site			0.827
C3–4	4	2	
C4–5	3	5	
C5–6	24	26	
C6–7	2	2	
Preoperative JOA scores	12.46 ± 1.61	12.07 ± 2.34	0.830
Preoperative NDI scores	15.67 ± 5.29	16.46 ± 6.45	0.584
Preoperative VAS scores	6.18 ± 2.48	6.09 ± 2.55	0.875
Preoperative Cobb C (°)	8.52 ± 3.16	9.40 ± 2.33	0.204
Preoperative Cobb S (°)	3.98 ± 1.49	3.50 ± 1.48	0.191
Preoperative TIH (cm)	3.18 ± 0.21	3.13 ± 0.16	0.316

*JOA* Japanese Orthopaedic Association, *NDI* Neck Disability Index, *VAS* visual analogue scale, *Cobb C* cervical alignment, *Cobb S* segmental angle, *TIH* total interbody height

### Surgical technique

After general anaesthesia, the patient was placed in a supine position with slight neck extension. After verification of the affected cervical disc level by fluoroscopy, a transverse incision was made on the right neck. Blunt dissection and retraction were performed between the trachea–oesophageal and carotid sheaths until the anterior vertebral body was exposed. After installation of the vertebral distractor, standard anterior discectomy was performed, and the posterior longitudinal ligament was opened for adequate decompression of the dura and the nerve roots. The residual cartilage tissue was thoroughly scraped until osseous bleeding of the endplate. According to the height and depth of the intervertebral space, the appropriate size of implants was confirmed by trial implants and fluoroscopy. For the Cage group, the cage was implanted into the intervertebral space, and an appropriate anterior cervical plate was fixed. For the Zero-P group, the stand-alone zero-profile devices were inserted. Finally, the incision was closed after placement of a drainage tube.

### Postoperative aftercare

Prophylactic intravenous antibiotics were administered 24 h after the operation, and drainage was maintained for 24–48 h and then removed. Patients were guided to arise from bed with a neck brace on the first postoperative day; at the same time, the patients were encouraged to perform functional exercises of the neck, shoulders, and back muscles. After discharge, a neck brace was used for 6 weeks.

### Clinical evaluations

The operation time and intraoperative blood loss were recorded. All patients were evaluated with the visual analogue scale (VAS) [11], Japanese Orthopaedic Association (JOA) scores [12], and Neck Disability Index (NDI) score [13] preoperatively and at 3 and 12 months postoperatively. Complications such as dysphagia, screw looseness or detachment, and adjacent segment degeneration were also recorded. The presence of dysphagia was assessed with the system designed by Bazaz [5] at 1 week, 3 months and 12 months postoperatively (Table 2).

**Table 2:** Bazaz grading system for dysphagia

Symptom severity	Liquid food	Solid food
None	None	None
Mild	None	Rare
Moderate	None or rare	Occasionally (only with specific food)
Severe	None or rare	Frequent (majority of solids)

### Radiographic evaluations

**Fig. 1:**
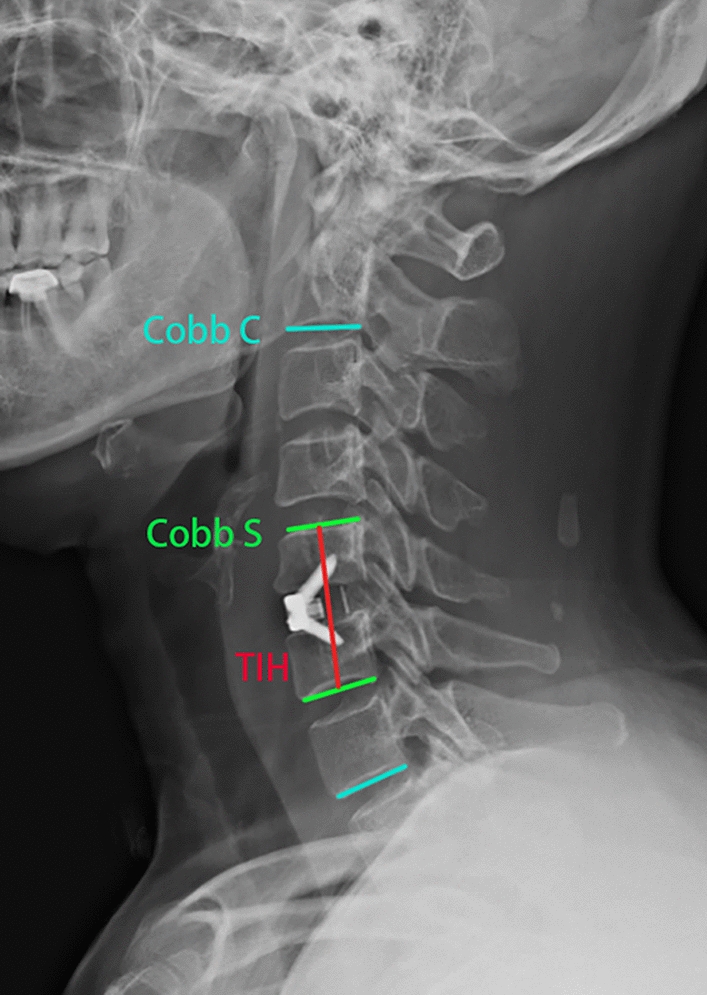
Lateral X-rays of the cervical vertebra. Cobb C: the Cobb angle between the lines perpendicular to the upper end plate of the C2 vertebral body and the lower end plate of the C7 vertebral body. Cobb S: the angle between a line perpendicular to the superior border of the upper affected vertebral body and the inferior border of the lower affected vertebral body. TIH: total interbody height

All patients underwent preoperative CT and MRI examinations. Anteroposterior and lateral X-rays of the neck in the neutral position and flexion–extension position were performed before the operation and at 1 week, 3 months and 12 months postoperatively. Cervical alignment (Cobb C) was calculated by the Cobb angle between the lines perpendicular to the upper end plate of the C2 vertebral body and the lower end plate of the C7 vertebral body. The segmental angle (Cobb S) was measured by the angle between a line perpendicular to the superior border of the upper affected vertebral body and the inferior border the lower affected vertebral body (Fig. 1). Cobb C and Cobb S were obtained preoperatively and at 3 and 12 months postoperatively to evaluate the aggravation of kyphosis [14]. The criteria of bone fusion were defined as follows: (a) less than 10° of motion on flexion/extension films; (b) presence of trabecular bridging at the bone-graft interface; (c) less than 50% radiolucency around the cage [14].

The total interbody height (TIH) of the segmental level was measured preoperatively and at 3 and 12 months postoperatively to assess the subsidence of implants [15]. The TIH was identified as the length from the midpoint of the upper end plate of the vertebral body to the lower end plate of the vertebral body on lateral X-ray views (Fig. 1). Subsidence occurred when the TIH in the final assessment decreased > 2 mm from 1 week postoperatively on lateral X-ray views. Additionally, we also recorded the number of patients with bony spur formation, an indication of degenerative changes of the adjacent segment.

### Statistical analysis

All data were analysed with Statistical Package for the Social Sciences software (SPSS 24.0, IBM, New York, NY, USA). Continuous data are expressed as the mean ± standard deviation (SD), and categorical data were tabulated with frequencies or percentages. Normality was tested using the Kolmogorov–Smirnov test. The independent *t*-test or the Mann–Whitney test was used to compare continuous data between two groups. The Chi-square test or Fisher’s exact test was used to analyse the categorical variables. A *P* value < 0.05 was considered statistically significant.

## Results

### Surgical outcomes

The mean operative time and blood loss in the two groups are shown in Table 3. Although the mean operative time and mean blood loss in the Zero-P group were lower than Cage group, obvious differences were observed only for operative time (*P* < 0.05). After surgery, all patients had primary incision healing, and no seroma or haematoma formation was detected in either group.

**Table 3:** Clinical outcomes

	Cage group (*n* = 33)	Zero-P group (*n* = 35)	*P* value
Operation time (min)	102.03 ± 33.6	85.11 ± 20.24	0.015
Blood loss (mL)	67.9 ± 95.6	30.8 ± 72.2	0.280
JOA			
Postoperative 3 months	14.55 ± 0.83	14.74 ± 1.12	0.415
Postoperative 12 months	15.61 ± 0.97	15.97 ± 0.86	0.103
NDI			
Postoperative 3 months	7.52 ± 3.78	7.77 ± 5.88	0.832
Postoperative 12 months	4.64 ± 3.81	4.03 ± 4.20	0.535
VAS			
Postoperative 3 months	2.27 ± 1.13	2.17 ± 1.65	0.770
Postoperative 12 months	0.79 ± 0.93	0.63 ± 0.88	0.469
Dysphagia			
Postoperative 1 week	7 (21.2%)	5 (14.3%)	0.534
Postoperative 3 months	5 (15.2%)	0 (0%)	0.023
Postoperative 12 months	5 (15.2%)	0 (0%)	0.023

*JOA* Japanese Orthopaedic Association, *NDI* Neck Disability Index, *VAS* visual analogue scale

### Clinical outcomes

The postoperative JOA, VAS and NDI scores at 3 months and 12 months postoperatively were higher than their respective preoperative values in both groups. Between the two groups, significant differences were not found for JOA, NDI or VAS at 3 months and 12 months postoperatively (*P* > 0.05). At 1 week postoperatively, five patients in the Zero-P group complained of mild dysphagia, and seven patients in the Cage group complained of mild (*n* = 6) or moderate (*n *= 1) dysphagia (*P* > 0.05). At 3 months postoperatively, no patients in the Zero-P group had dysphagia. In the Cage group, five patients had mild dysphagia at 3 months postoperatively, and these five patients still had mild dysphagia at 12 months postoperatively. The occurrence of dysphagia was significantly different between the two groups at 3 months and 12 months postoperatively (*P* < 0.05) (Table 3).

### Radiographic outcomes

Compared with the preoperative values, the Cobb C and Cobb S values were improved for both groups at 3 months and 12 months postoperatively. In the Cage group, the Cobb C was 13.91 ± 4.44° at 3 months postoperatively, which decreased to 11.40 ± 3.19° at 12 months postoperatively, while the Cobb C was 14.83 ± 4.35° and 12.66 ± 3.17° in the Zero-P group at 3 months and 12 months postoperatively, respectively. The Cobb S of the Cage group increased from 6.68 ± 2.65° at 3 months after the operation to 7.08 ± 2.36° at 12 months postoperatively. In the Zero-P group, Cobb S increased from 6.11 ± 2.27° at the 3-month postoperative assessment to 7.19 ± 1.74° at 12 months postoperatively. No significant difference was observed between the two groups for either Cobb C or Cobb S at any time after surgery. Regarding the extent of the TIH, the two groups had a significant increase in the distance between the vertebral bodies at 3 months and 12 months after the operation. No significant differences were observed between the two groups for the TIH at any time after surgery. Based on observation of the TIH, subsidence occurred in five patients in the Cage group and four patients in the Zero-P group at 12 months postoperatively. The occurrence rate of subsidence in the two groups was not significantly different (*P* > 0.05) (Table 4).

**Table 4:** Radiographic outcomes

	Cage group (*n* = 33)	Zero-P group (*n* = 35)	*P* value
Cobb C (°)			
Postoperative 3 months	13.91 ± 4.44	14.83 ± 4.35	0.467
Postoperative 12 months	11.40 ± 3.19	12.66 ± 3.17	0.122
Cobb S (°)			
Postoperative 3 months	6.68 ± 2.65	6.11 ± 2.27	0.344
Postoperative 12 months	7.08 ± 2.36	7.19 ± 1.74	0.818
TIH (cm)			
Postoperative 3 months	3.50 ± 0.30	3.44 ± 0.25	0.354
Postoperative 12 months	3.42 ± 0.15	3.36 ± 0.23	0.271
Subsidence	5 (15.2%)	4 (11.4%)	0.730
Fusion (%)	32 (97.0%)	35 (100%)	0.485
Bony spurs (%)	5 (15.2%)	0 (0%)	0.023

*Cobb C* cervical alignment, *Cobb S* segmental angle, *TIH* total interbody height

**Fig. 2:**
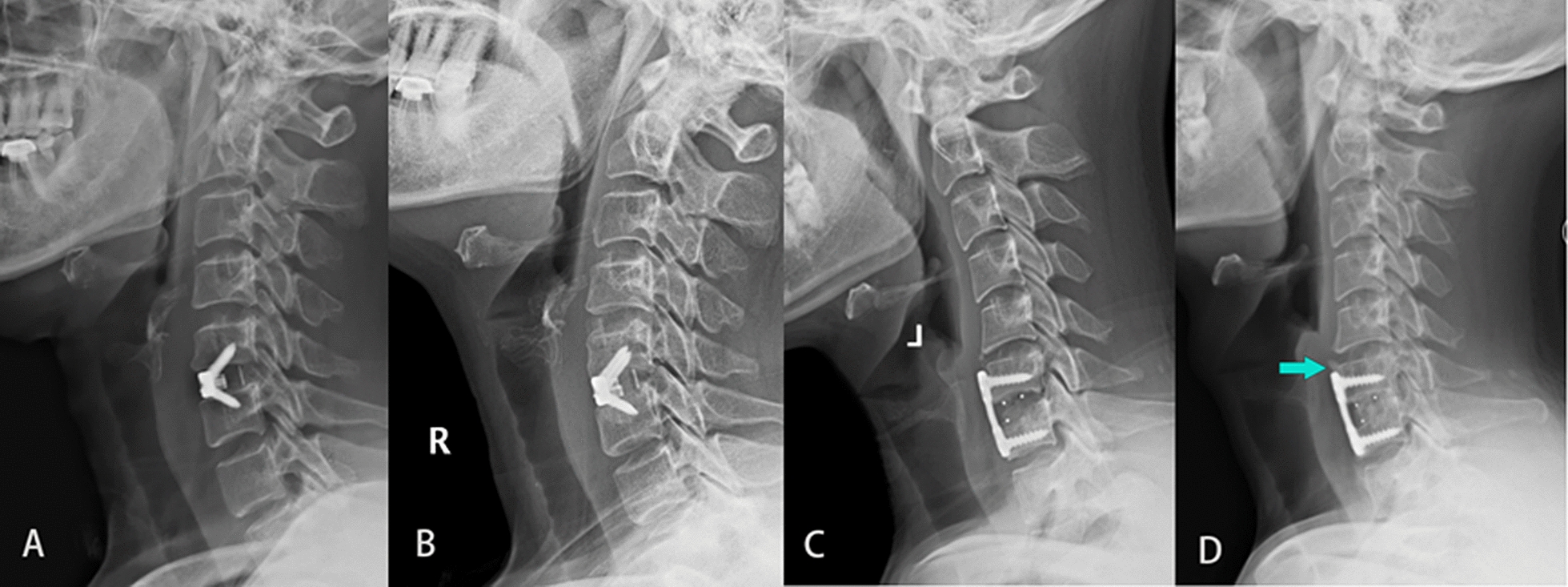
**A **and** C** Lateral X-ray image of the cervical spine at 3 months postoperatively. **B **and** D** Lateral X-ray images of the cervical spine at 12 months postoperatively. A new anterior bony spur has formed from the tip of the plate to the upper adjacent level, as shown in 2D

During the follow-up, we did not observe screw looseness or screw detachment. Bone fusion was found in 97% and 100% of the Cage and Zero-P groups, respectively. No significant difference was detected in the fusion rate between the two groups (*P* > 0.05). The numbers of patients with bony spurs were five in the Cage group and none in the Zero-P group. The occurrence rate of bony spurs was significantly different between the two groups (*P* < 0.05) (Table 4). Series radiographs of typical cases are shown in Fig. 2.

## Discussion

ACDF is the standard surgical treatment for cervical spondylosis when conservative treatment fails [16]. With increasing experience, an increasing number of reports have confirmed the effectiveness of additional plates in the treatment of degenerative spinal diseases [4, 17, 18]. It was demonstrated that plates resulted in a higher fusion rate and immediate postoperative stability and decreased the occurrence of pseudarthrosis [19, 20]. In addition, it also prevented interbody cage dislocation and subsidence, which might occur after multilevel procedures [21]. Although this treatment has been widely accepted, it also has several drawbacks, one of which is dysphagia postoperatively [22–24].

Chronic dysphagia is one of the common complications after ACDF. The reported incidence of this complication has ranged from 3 to 21% [5, 9, 25, 26]. There are several potential reasons for dysphagia, such as soft tissue swelling, haematoma, oesophageal injury, and adhesion formation around implanted cervical plates [27]. Adjacent segment degeneration is another complication of plate implantation [28]. To solve these problems, a stand-alone cage was supposed to be a good alternative and was proven to bring satisfying outcomes [24, 29]. However, stand-alone cages increased the occurrence rate of implant subsidence and caused secondary kyphotic deformity, constituting a potential reason for adjacent cervical segment disease in the long term [30]. Therefore, cage screw devices that can provide immediate biomechanical stability, such as anterior cervical plates, have been developed (e.g., Zero-P by DePuy Synthes).

The entirety of the zero-profile anchored cage consists of a plate and screw system, eliminating the basic disadvantage of stand-alone cages, which is extension instability [15, 24, 31]. It was reported that either the zero-profile anchored cage or a traditional titanium plate with a cage has a satisfying effect on improving the symptoms of cervical degeneration disease. However, less dysphagia has been observed in the Zero-P group [10, 32]. Furthermore, it can be inferred that the utilization of zero-P anchored cage can reduce spondylopathy in adjacent segments, and the underlying mechanism is that the zero-profile anchored cage transmits less stress to adjacent segments. Other studies have come to similar conclusions [9, 10]. In this study, we also found that the incidence of dysphagia was lower in the Zero-P group at 3 and 12 months after the operation. In conclusion, we find that the zero-profile anchored cage can reduce the incidence of dysphasia.

There was significant improvement in neurological function and symptoms in both groups according to clinical efficacy assessed by VAS, JOA and NDI. The symptoms of cervical spondylosis are due to stimulation or compression of degenerative intervertebral discs. Degenerative intervertebral discs were removed thoroughly in both groups. Therefore, both zero-profile anchored cage and traditional cage-plate fixation can bring good clinical results.

Adjacent segmental degeneration is confirmed when there is height reduction, instability, or osteophyte formation in the adjacent intervertebral space. Fusion of surgical segments leads to increased stress in adjacent segments [33, 34]. In addition, the application of the anterior plate stimulates the adjacent segments, leading to the formation and degeneration of bone spurs [35]. Based on the observation of lateral X-rays, there were no patients in the Zero-P group, while 4 patients in the Cage group developed bony spurs at 12 months postoperatively in this study. The difference was significant.

Several limitations existed in this study. One of the major limitations was that it was a retrospective study. A randomized, controlled study is needed to further investigate the efficacy of the zero-profile anchored cage and traditional cage-plate fixation in single-level anterior cervical discectomy and fusion. Second, the length of follow-up in this study was relatively insufficient, and a longer follow-up is required to compare the long-term effects of the zero-profile anchored cage and traditional cage-plate fixation in the treatment of cervical spondylosis. Furthermore, since all of the patients in this study were treated by one surgical team at a single centre, multicentre research is needed to further verify our conclusions.

## Conclusions

The results of this study showed good results for both zero-profile anchored cage and traditional cage-plate fixation for cervical degenerative disc disease in terms of improvement in clinical outcomes and radiographic outcomes. We concluded that either the zero-profile anchored cage or traditional cage-plate fixation is effective treatment of single-level cervical spondylosis. However, the zero-profile anchored cage might be associated with a lower incidence of adjacent segment degeneration and dysphagia. In addition, the zero-profile anchored cage is associated with a shorter operation time and relatively greater simplicity than the traditional cage-plate fixation. Therefore, the zero-profile anchored cage is a good substitute for traditional cage-plate fixation.

## Data Availability

The datasets used and/or analysed during the current study are available from the corresponding author on reasonable request.

## References

[1] Karadimas SK, Erwin WM, Ely CG (2013). Pathophysiology and natural history of cervical spondylotic myelopathy. Spine.

[2] Yamazaki T, Yanaka K, Sato H (2003). Cervical spondylotic myelopathy: surgical results and factors affecting outcome with special reference to age differences. Neurosurgery.

[3] Petr V, Ondrej B, Patricia D (2013). Anterior interbody fusion of the cervical spine with Zero-P spacer: prospective comparative study—clinical and radiological results at a minimum 2 years after surgery. Spine.

[4] Song KJ, Lee K, Taghavi CE (2009). The efficacy of plate construct augmentation versus cage alone in anterior cervical fusion. Spine.

[5] Bazaz R, Lee MJ, Yoo JU (2002). Incidence of dysphagia after anterior cervical spine surgery: a prospective study. Spine.

[6] Hwang SL, Lin CL, Lieu AS (2004). Three-level and four-level anterior cervical discectomies and titanium cage-augmented fusion with and without plate fixation. J Neurosurg Spine.

[7] Riley LH, Skolasky RL, Albert TJ (2005). Dysphagia after anterior cervical decompression and fusion: prevalence and risk factors from a longitudinal cohort study. Spine.

[8] Azab W, Abdel-Razek M, Ali A (2012). Outcome evaluation of a zero-profile implant for anterior cervical diskectomy with fusion. Turk Neurosurg.

[9] Scholz M, Schnake KJ, Pingel A (2011). A new zero-profile implant for stand-alone anterior cervical interbody fusion. Clin Orthop Relat Res.

[10] Wang Z, Zhu R, Yang H (2014). The application of a zero-profile implant in anterior cervical discectomy and fusion. J Clin Neurosci.

[11] Huskisson E (1974). Measurement of pain. Lancet.

[12] Wang S, Wang B, Yu X (2021). Efficacy of gelatin sponge impregnated with ropivacaine on postoperative pain after transforaminal lumbar interbody fusion: a comparative study. Bmc Musculoskel Dis.

[13] Scholz M, Onal B, Schleicher P (2020). Two-level ACDF with a zero-profile stand-alone spacer compared to conventional plating: a prospective randomized single-center study. Eur Spine J.

[14] Cho HJ, Hur JW, Lee JB (2015). Cervical stand-alone polyetheretherketone cage versus zero-profile anchored spacer in single-level anterior cervical discectomy and fusion : minimum 2-year assessment of radiographic and clinical outcome. J Korean Neurosurg S.

[15] Kandziora F, Pflugmacher R, Sch Fer J (2001). Biomechanical comparison of cervical spine interbody fusion cages. Spine.

[16] Li J, Zheng Q, Guo X (2013). Anterior surgical options for the treatment of cervical spondylotic myelopathy in a long-term follow-up study. Arch Orthop Traum Su.

[17] Fraser JF, Härtl R (2007). Anterior approaches to fusion of the cervical spine: a metaanalysis of fusion rates. J Neurosurg Spine.

[18] Troyanovich SJ, Stroink AR, Kattner KA (2002). Does anterior plating maintain cervical lordosis versus conventional fusion techniques? A retrospective analysis of patients receiving single-level fusions. J Spinal Disord Tech.

[19] Anderson DG, Albert TJ (2002). Bone grafting, implants, and plating options for anterior cervical fusions. Orthop Clin N Am.

[20] Bohler J, Gaudernak T (1980). Anterior plate stabilization for fracture—dislocations of the lower cervical spine. J Trauma.

[21] Pitzen TR, Chrobok J, Tulik J (2009). Implant complications, fusion, loss of lordosis, and outcome after anterior cervical plating with dynamic or rigid plates: two-year results of a multi-centric, randomized. Control Study Spine.

[22] Fernández-Fairen M, Sala P, Dufoo M (2008). Anterior cervical fusion with tantalum implant. Spine.

[23] Hacker RJ, Cauthen JC, Gilbert TJ (2000). A prospective randomized multicenter clinical evaluation of an anterior cervical fusion cage. Spine.

[24] Peolsson A, Vavruch L, Hedlund R (2007). Long-term randomised comparison between a carbon fibre cage and the Cloward procedure in the cervical spine. Eur Spine J.

[25] Kalb S, Reis MT, Cowperthwaite MC (2012). Dysphagia after anterior cervical spine surgery: incidence and risk factors. World Neurosurg.

[26] Lee MJ, Bazaz R, Furey CG (2005). Influence of anterior cervical plate design on Dysphagia: a 2-year prospective longitudinal follow-up study. J Spinal Disord Tech.

[27] Fountas KN, Kapsalaki EZ, Nikolakakos LG (2007). Anterior cervical discectomy and fusion associated complications. Spine.

[28] Kao FC, Niu CC, Chen LH (2005). Maintenance of interbody space in one- and two-level anterior cervical interbody fusion: comparison of the effectiveness of autograft, allograft, and cage. Clin Orthop Relat Res.

[29] Hedlund R, Leszniewski W, Vavruch L (2002). A prospective randomized comparison between the Cloward procedure and a carbon fiber cage in the cervical spine: a clinical and radiologic study. Spine.

[30] Gercek E, Arlet V, Delisle J (2003). Subsidence of stand-alone cervical cages in anterior interbody fusion: warning. Eur Spine J.

[31] Matgé G (2002). Cervical cage fusion with 5 different implants: 250 cases. Acta Neurochir.

[32] Xiao SW, Liang ZD, Wei W (2017). Zero-profile anchored cage reduces risk of postoperative dysphagia compared with cage with plate fixation after anterior cervical discectomy and fusion. Eur Spine J.

[33] Ec KJC, Humphreys SC, Lim TH (2002). Biomechanical study on the effect of cervical spine fusion on adjacent-level intradiscal pressure and segmental motion. Spine.

[34] Park DH, Ramakrishnan P, Cho TH (2007). Effect of lower two-level anterior cervical fusion on the superior adjacent level. J Neurosurg Spine.

[35] Yue WM, Brodner W, Highland TR (2004). Persistent swallowing and voice problems after anterior cervical discectomy and fusion with allograft and plating: a 5- to 11-year follow-up study. Spine J.

